# Somatostatin and Its Receptors in Myocardial Ischemia/Reperfusion Injury and Cardioprotection

**DOI:** 10.3389/fphar.2021.663655

**Published:** 2021-11-05

**Authors:** Imre Vörös, Éva Sághy, Krisztina Pohóczky, András Makkos, Zsófia Onódi, Gábor B. Brenner, Tamás Baranyai, Bence Ágg, Barnabás Váradi, Ágnes Kemény, Przemyslaw Leszek, Anikó Görbe, Zoltán V. Varga, Zoltán Giricz, Rainer Schulz, Zsuzsanna Helyes, Péter Ferdinandy

**Affiliations:** ^1^ Cardiometabolic Research Group and MTA-SE System Pharmacology Research Group, Department of Pharmacology and Pharmacotherapy, Semmelweis University, Budapest, Hungary; ^2^ HCEMM-SU Cardiometabolic Immunology Research Group, Budapest, Hungary; ^3^ Department of Pharmacology, Faculty of Pharmacy, University of Pécs, Pécs, Hungary; ^4^ Szentágothai János Research Center, University of Pécs, Pécs, Hungary; ^5^ Department of Pharmacology and Pharmacotherapy, Medical School, University of Pécs, Pécs, Hungary; ^6^ Pharmahungary Group, Szeged, Hungary; ^7^ Heart and Vascular Center, Semmelweis University, Budapest, Hungary; ^8^ Department of Medical Biology, University of Pécs, Pécs, Hungary; ^9^ Department of Heart Failure and Transplantology, Cardinal Stefan Wyszyński National Institute of Cardiology, Warszawa, Poland; ^10^ Institute of Physiology, Justus-Liebig-University Giessen, Giessen, Germany

**Keywords:** somatostatin, somatostatin receptor, ischemia-reperfusion, myocardial infarction, ischemic conditioning, translational research, gene expression

## Abstract

Little is known about the role of the neuropeptide somatostatin (SST) in myocardial ischemia/reperfusion injury and cardioprotection. Here, we investigated the direct cardiocytoprotective effect of SST on ischemia/reperfusion injury in cardiomyocyte cultures, as well as the expression of SST and its receptors in pig and human heart tissues. SST induced a bell-shaped, concentration-dependent cardiocytoprotection in both adult rat primary cardiomyocytes and H9C2 cells subjected to simulated ischemia/reperfusion injury. Furthermore, in a translational porcine closed-chest acute myocardial infarction model, ischemic preconditioning increased plasma SST-like immunoreactivity. Interestingly, SST expression was detectable at the protein, but not at the mRNA level in the pig left ventricles. *SSTR1* and *SSTR2*, but not the other SST receptors, were detectable at the mRNA level by PCR and sequencing in the pig left ventricle. Moreover, remote ischemic conditioning upregulated *SSTR1* mRNA. Similarly, SST expression was also detectable in healthy human interventricular septum samples at the protein level. Furthermore, SST-like immunoreactivity decreased in interventricular septum samples of patients with ischemic cardiomyopathy. *SSTR1, SSTR2,* and *SSTR5* but not *SST* and the other SST receptors were detectable at the mRNA level by sequencing in healthy human left ventricles. In addition, in healthy human left ventricle samples, *SSTR1* and *SSTR2* mRNAs were expressed especially in vascular endothelial and some other cell types as detected by RNA Scope^®^ *in situ* hybridization. This is the first demonstration that SST exerts a direct cardiocytoprotective effect against simulated ischemia/reperfusion injury. Moreover, SST is expressed in the heart tissue at the peptide level; however, it is likely to be of sensory neural origin since its mRNA is not detectable. SSTR1 and SSTR2 might be involved in the cardioprotective action of SST, but other mechanisms cannot be excluded.

## Introduction

Ischemic heart disease belongs to the leading causes of death worldwide (Hausenloy et al., 2017). There is no effective cardioprotective drug therapy on the market to reduce tissue injury; however, the heart has adaptive mechanisms, e.g., ischemic preconditioning, ischemic postconditioning, and remote ischemic conditioning, which can decrease the tissue damage caused by a subsequent ischemic insult (Ferdinandy et al., 2014; Bencsik et al., 2020). These conditioning mechanisms are short, repeated ischemia/reperfusion cycles applied either on the myocardium or on a remote organ before, during, or after an ischemic insult (Ferdinandy et al., 2014). The underlying mechanisms and mediators are still not fully clarified (Davidson et al., 2019).

Sensory nerves innervating the heart are involved in cardiac adaptation mechanisms to ischemic injury (Bencsik et al., 2020; Szabados et al., 2020). The activation of the Transient Receptor Potential Vanilloid 1- (TRPV1-) expressing capsaicin-sensitive chemosensitive peptidergic afferents leads to the release of sensory neuropeptides (Holzer, 1988). The role of the calcitonin gene-related peptide (CGRP) and substance P (SP) in myocardial protection was extensively investigated. Several studies suggest that CGRP and SP mediate the cardioprotective effect of ischemic preconditioning, ischemic postconditioning, and remote ischemic conditioning (Lu et al., 1999; Chai et al., 2006; Zhong and Wang, 2007; Ren et al., 2011; Gao et al., 2015; Randhawa and Jaggi, 2017; Guo et al., 2018). Besides proinflammatory neuropeptides, inhibitory mediators, e.g., pituitary adenylate cyclase-activating polypeptide (PACAP), exerts a direct cytoprotective effect on neonatal rat cardiomyocytes against ischemia/reperfusion-induced apoptosis (Roth et al., 2009). Furthermore, somatostatin (SST) released from these sensory nerves might also be involved in cardioprotection (Holzer, 1988; Helyes et al., 2000).

SST is a small peptide that is also released from the central nervous system besides the sensory nerves (Helyes et al., 2009), from inflammatory and immune cells, pancreas, retinal neurons, and epithelial cells (Rai et al., 2015). SST exerts anti-inflammatory, antinociceptive (Helyes et al., 2009; Markovics et al., 2012), antisecretory, and antiproliferative effects (Rai et al., 2015). There are five G_i_-protein-coupled transmembrane receptor subtypes of SST (SSTR1-5) (Rai et al., 2015). It is known that endogenous SST released from capsaicin-sensitive nerves prevented retinal ischemia/reperfusion injury in a mouse model (Wang et al., 2017). Exogenous application of synthetic SST analogs exerts protective effects against ischemia/reperfusion injury of rat and mouse retina (Kokona et al., 2012; Wang et al., 2015), rabbit liver (Yang et al., 2013), rat pancreas (Hoffmann et al., 1996), and rat heart (Wang et al., 2005). However, little is known about SST expression and function in ischemia/reperfusion injury of the heart and its involvement in cardioprotection.

Therefore, here, we aimed to investigate the direct cardiocytoprotective effect of SST against simulated ischemia/reperfusion injury in cardiac cell cultures, as well as the expression of SST and its receptors in pig and human hearts.

## Materials and Methods

### Cardiac Cell Cultures

H9C2 cell line originates from the European Collection of Authenticated Cell Cultures and it was purchased from Sigma-Aldrich (St. Louis, MO, United States). H9C2 cells were plated on 96-well plates (2*10^4^ cells/well) and incubated for 24 h in Dulbecco’s Modified Eagle’s Medium (Corning, NY, United States) supplemented with 10% fetal bovine serum (FBS) (Euroclone SpA. Milan/Stockholm).

The primary culture of adult rat cardiomyocytes was prepared as described previously (Makkos et al., 2019). Briefly, male Wistar rats (150–200 g) were anesthetized with pentobarbital (60 mg/kg) and heparinized (500 IU/kg). Hearts were excised and perfused with Krebs-Henseleit solution, followed by digestion with collagenase II (8000 U/mL) for 30–45 min. Ventricles were cut into small pieces and digested for 10 min. Cell suspension was filtered and pelleted under gravity. Ca^2+^ concentration was increased gradually up to a final of 1 mM. Cells (7,500 cells/well) were plated on laminin-coated glass coverslips in a 24-well plate (Thermo Fisher Scientific, Waltham, MA, United States) and maintained in proliferation media for 3 h (5% FBS containing M199) and in growth media (serum-free M199) for 24 h.

### *In Vitro* Simulated Ischemia/Reperfusion Injury Study Protocol and Cell Viability Measurement

**FIGURE 1:**
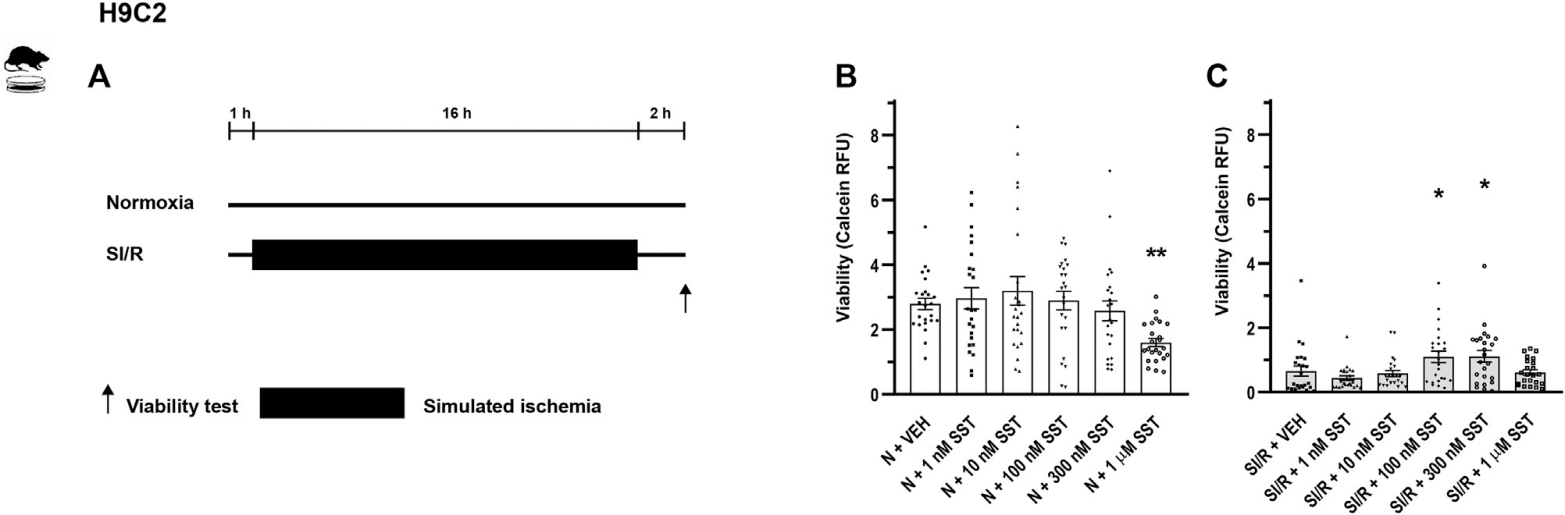
*In vitro* simulated ischemia/reperfusion (SI/R) study protocol on H9C2 cell line **(A)**. Somatostatin (SST) (1 nM, 10 nM, 100 nM, and 300 nM and 1 µM) or its vehicle (VEH) was added to the cells during the whole experiment. Viability of SST-treated H9C2 cells exposed to normoxia (N) **(B)** or SI/R **(C)**. Data are presented as mean ± SEM. The Kruskal-Wallis test and Dunn’s post hoc test, *p < 0.05; **p < 0.01 vs. VEH from four independent experiments. RFU: relative fluorescence unit.

**FIGURE 2:**
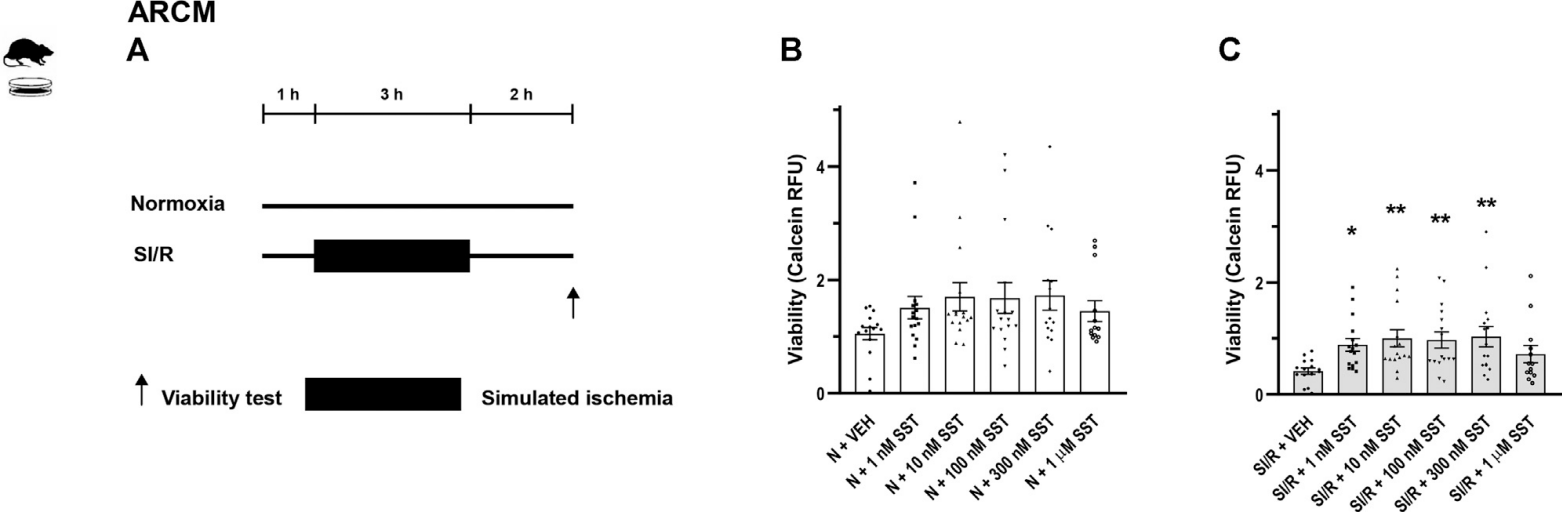
*In vitro* simulated ischemia/reperfusion (SI/R) study protocol on isolated adult rat cardiomyocytes (ARCM) **(A)**. Somatostatin (SST) (1 nM, 10 nM, 100 nM, and 300 nM and 1 µM) or its vehicle (VEH) was added to the cells during the whole experiment. Viability of SST-treated ARCM cells exposed to normoxia (N) **(B)** or SI/R **(C)**. Data are presented as mean ± SEM. The Kruskal-Wallis test and Dunn’s post hoc test, *p < 0.05; **p < 0.01 vs. VEH from four independent experiments. RFU: relative fluorescence unit.

Study protocols are demonstrated in Figures 1A, 2A. The concentrations of SST were selected for the *in vitro* experiments on the basis of competition binding and G-protein activation results on CHO cells (Markovics et al., 2012). Cells were treated with either SST (1, 10, 100, and 300 nM and 1 µM) or its vehicle in growth media in a CO_2_ incubator for 1 h. Then H9C2 cells and adult rat cardiomyocytes were covered either with normoxic solution (in mM: NaCl 125, KCl 5.4, NaH_2_PO_4_ 1.2, MgCl_2_ 0.5, HEPES 20, CaCl_2_ 1, glucose 15, taurine 5, creatine-monohydrate 2.5, bovine serum albumin (BSA) 0.1%, and pH 7.4) in CO_2_ incubator (normoxia groups) or with hypoxic solution (in mM NaCl 119, KCl 5.4, NaH_2_PO_4_ × 1H_2_O 1.2, MgCl_2_ × 6H_2_O 0.5, HEPES 5, MgSO_4_ × 7H_2_O 1.3, CaCl_2_ × 2H_2_O 0.9, Na-lactate 20, BSA 0.1%, and pH 6.4) in a three-gas (mixture of 95% N_2_ and 5% CO_2_) incubator (simulated ischemia/reperfusion groups), both solutions containing SST or its vehicle for 16 and 3 h, respectively. Then, during the simulated reperfusion phase, cells were kept in growth media containing vehicle or SST in a CO_2_ incubator for 2 h. Cell viability was determined using calcein staining (Makkos et al., 2019). After rinsing with Dulbecco’s phosphate-buffered saline (D-PBS), cells were incubated with calcein solution (1 µM) for 30 min at room temperature. Fluorescence intensity of each well was detected by Varioskan Lux multimode microplate reader (Thermo Fisher Scientific, Waltham, MA, United States) at room temperature; excitation wavelength: 490 nm; emission wavelength: 520 nm. Four independent experiments were performed (n = 4) averaging at 70% confluence of cell cultures. Regarding H9C2 cells, a 96-well format was applied and an average of six-well relative fluorescence units (RFU) was used in one treatment group. Regarding adult rat cardiomyocytes, a 24-well format was applied and an average of four wells RFU was used in one treatment group. Results are shown as RFU (Brenner et al., 2020).

### Closed-Chest Pig Model of Acute Myocardial Infarction and Tissue Sampling

**FIGURE 3:**
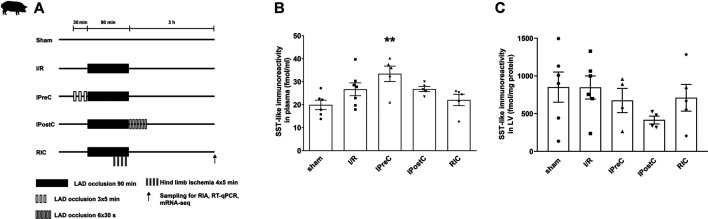
Closed-chest porcine acute myocardial infarction study protocol **(A)**. Somatostatin-like immunoreactivity of plasma **(B)** and left ventricular samples **(C)**. Data are presented as mean ± SEM. **p < 0.01 vs. sham (n = 6), one-way ANOVA, Tukey post hoc test, I/R: ischemia-reperfusion (n = 7 in plasma and 6 in LV), IPreC: ischemic preconditioning (n = 5 in plasma and 4 in LV), IPostC: ischemic postconditioning (n = 5 in plasma and 4 in LV), RIC: remote ischemic conditioning (both n = 5), LAD: left anterior descending coronary artery, and LV: left ventricle.

Porcine left ventricle and plasma samples of the present study have been obtained from one of our previous studies in a closed-chest pig model of acute myocardial infarction and cardioprotection by ischemic pre-, post-, and remote conditioning (Baranyai et al., 2017). The study was conducted according to the Guide for the Care and Use of Laboratory Animals published by the US National Institutes of Health (NIH publication No. 85–23, revised 1996) and to the EU Directive (2010/63/EU) and was reported according to the ARRIVE guidelines (Kilkenny et al., 2010). The study was approved by the Animal Ethics Committee of the Hungarian National Food Chain Safety Office (SOI/31/26–11/2014). Briefly, pigs were randomly divided into five groups: ischemia/reperfusion, ischemic preconditioning, ischemic postconditioning, remote ischemic conditioning, and sham-operated groups. In the ischemia/reperfusion group, 90 min of myocardial ischemia was applied, which was induced by left anterior descending coronary artery (LAD) occlusion. The ischemic preconditioning group was subjected to 3 × 5 min myocardial ischemia prior to 90 min LAD occlusion. The ischemic postconditioning group was subjected to 6 × 30 s of myocardial ischemia after 90 min LAD occlusion at the start of the reperfusion. The remote ischemic conditioning group was subjected to 4 × 5 min of hind limb ischemia during the 90 min LAD occlusion. In the sham group, the balloon catheter was inserted in the LAD coronary artery, but it was not inflated (Figure 3A). After 3 h reperfusion, plasma samples and myocardial tissue samples were collected from the ischemic region of the left ventricle myocardium. Group sizes are as follows: sham (n = 6–7), ischemia/reperfusion (n = 5–7), ischemic preconditioning (n = 4–5), ischemic postconditioning (n = 4–5), and remote ischemic conditioning (n = 4–5). Samples were frozen immediately and stored at −80°C. Infarct size measurements confirmed that ischemic preconditioning decreases myocardial necrosis significantly compared to ischemia/reperfusion after 3 h of reperfusion. There was no difference in the area at risk between groups. Ischemic postconditioning and remote ischemic conditioning decrease edema significantly compared to ischemia/reperfusion in the myocardium after 3 days of reperfusion, and ischemic preconditioning shows only a tendency of decrease. For details of the phenotype of the pig model, see (Baranyai et al., 2017).

### Human Heart Tissue Collection

All experiments were designed and implemented according to the ethical standards of the Declaration of Helsinki (1975). Patients gave their written informed consent to be involved in the study. The protocol was approved in 2018 by the Polish Local Ethics Committee of the National Institute of Cardiology in Warsaw with the identification code IK-NPIA-0021-14/1426/18 project 4.

Human left ventricle and interventricular septum samples were collected in the Department of Heart Failure and Transplantology, Cardinal Stefan Wyszyński National Institute of Cardiology, Warszawa, Poland, as previously described (Varga et al., 2017). Human hearts not used for transplantation for various reasons were obtained from organ donors (control group, CON, *n*_*female*_ = 6 and n_male_ = 4). The donors with any relevant previous cardiovascular history or any abnormalities in ECG and echocardiography were not included in the present study. Failing hearts were obtained from patients suffering from advanced heart failure of ischemic cardiomyopathy (ICM, *n*_*female*_ = 1 and *n*_*male*_ = 9). Detailed clinical parameters of the patients are summarized in Figure 5.

Left ventricle and interventricular septum samples were collected during heart explantation, avoiding the inclusion of scarred/fibrotic or adipose tissues, endocardium, epicardium, or coronary vessels. The samples were rinsed immediately in saline, blotted dry, frozen in liquid nitrogen, and kept at −80°C until processing for further molecular assays. Another series of left ventricle samples were fixed in neutral buffered formalin and embedded in paraffin for histological assays.

### SST-Like Immunoreactivity Measurement With Radioimmunoassay

Left ventricle tissue samples were homogenized in 8x volumes (in µL) of distilled water containing 10 µL protease inhibitor (Gordox, 10,000 KIE/mL, Gedeon Richter Plc) using a tissue homogenizer (IKA T25 Digital ULTRA TURRAX). Distilled water shows a similar protein extraction efficiency with pig heart tissue as described in the literature with a different buffer composition (Parés et al., 2020). Afterward, samples were centrifuged at 10,000 rpm at 4°C for 15 min. Protein was extracted from each 1 ml of the plasma with the mixture of 3 ml absolute ethanol and 10 µL 96% acetic acid. Tubes were incubated at room temperature for 30 min, followed by centrifugation at 3,000 rpm for 20 min at 4°C. The supernatant was collected into a reaction tube and dried under a nitrogen flow for 6 h at room temperature. Then, it was resuspended in 300 μl assay buffer and centrifuged at 10,000 rpm at 4°C for 15 min before radioimmunoassay (RIA) determination.

SST-like immunoreactivity was determined by a specific and sensitive RIA method developed in our laboratory as described earlier in detail (Nemeth et al., 1996). Briefly, each reaction tube contained 25 µL tissue homogenates or 750 µl suspended dry plasma sample, 100 µl ^125^I-labeled somatostatin-14 as tracer (3,000 cpm/tube), and 100 µl antiserum (1:445,000). Tubes were filled to 1,000 µl with RIA assay buffer. Antiserum has been raised in sheep against somatostatin-14-bovine thyroglobulin. Following 72 h incubation at 4°C, antigen-bound and free peptides were separated. Tubes were centrifuged at 4°C at 4,000 rpm for 20 min. Radioactivity was measured by a gamma counter (Gamma NZ-310, Hungary). The results were expressed as fmol SST-like immunoreactivity per mg total protein weight in the tissue and per mL in the plasma samples.

### Total RNA Isolation and Real-Time Quantitative PCR of Pig Heart Samples

Total RNA from pig left ventricle samples (ischemic zone) was extracted using Direct-Zol RNA Mini Prep (Zymo Research, Irvine, CA, United States) according to the manufacturer’s instructions. RNA was then treated with DNase I (Zymo Research, Irvine, CA, United States), and RNA concentration was determined by spectrophotometer (NanoDrop ND-1000, NanoDrop Technologies Inc., Wilmington, DE, United States). 1 µg of total RNA/sample was reverse transcribed into complementary DNA (cDNA) using Maxima™ First Strand cDNA Synthesis Kit for real-time quantitative PCR (RT-qPCR) (Thermo Scientific, Waltham, MA, United States).

The expression of somatostatin and its receptors was assessed using Biometria TProfessional Basic Gradient PCR equipment (Biometra GmbH, Göttingen, Germany). PCR products were identified by their size using 2% agarose gel electrophoresis.

Relative gene expression ratios were determined with Stratagene Mx3000P QPCR System (Agilent Technologies, Santa Clara, CA, United States) using β*-ACTIN* as a reference gene based on our pilot experiments and literature data (Nygard et al., 2007). Target genes were amplified using 1x Luminaris HiGreen Low ROX qPCR Master Mix (Thermo Scientific, Waltham, MA, United States). Amplifications were carried out under the following conditions: 95°C 10 min, followed by 40 cycles of 95°C 30 s, 60°C 30 s, and 72°C 45 s. Measurements included dissociation curve analysis to ensure amplification specificity. The corresponding group sizes are ischemia/reperfusion group (SSTR1: n = 5, SSTR2: n = 6), ischemic preconditioning group (n = 4), ischemic postconditioning group (n = 5), remote ischemic conditioning group (SSTR1: n = 5, SSTR2: n = 4), and sham group (n = 7). Primers and product lengths for each gene are listed in Supplementary Table S1.

### Total RNA Isolation and RNA-Sequencing of Human Heart Samples

Samples were collected and lysed in 1 ml of QIAzol Lysis Reagent (QIAGEN, Hombrechtikon, Switzerland). Total RNA was extracted from 350 µl of the lysates using Direct-Zol RNA Mini Prep System with on-column DNase I treatment according to the manufacturer’s (Zymo Research, Irvine, CA, United States) protocol. The RNA integrity numbers and RNA concentration were determined by RNA ScreenTape system with 2,200 Tapestation (Agilent Technologies, Santa Clara, CA, United States) and RNA HS Assay Kit with Qubit 3.0 Fluorometer (Thermo Fisher Scientific, Waltham, MA, United States), respectively.

For Gene Expression Profiling (GEx) library construction, QuantSeq 3’ mRNA-Seq Library Prep Kit FWD for Illumina (Lexogen GmbH, Wien, Austria) was applied according to the manufacturer’s protocol. The quality and quantity of the library were determined by using High Sensitivity DNA1000 ScreenTape system with 2,200 Tapestation (Agilent Technologies, Santa Clara, CA, United States) and dsDNA HS Assay Kit with Qubit 3.0 Fluorometer (Thermo Fisher Scientific, Waltham, MA, United States), respectively. Pooled libraries were diluted to 1.8 pM for 1 × 86 bp single-end sequencing with 75-cycle High Output v2.5 Kit on the NextSeq 500 Sequencing System (Illumina, San Diego, CA, United States) according to the manufacturer’s protocol. RNA-sequencing datasets of the human samples are stored in the ArrayExpress database with the following accession number: E-MTAB-10720. Out of this data set, here, we present the *SST* and *SSTR1-5* genes-related sequencing data obtained from control human cardiac samples.

### Analysis of mRNA-Sequencing Data

#### Pig Heart Samples

mRNA-sequencing data of left ventricle samples of pig myocardial infarction model (Lukovic et al., 2019) were analyzed in our present study. The detailed protocol of the mRNA-sequencing method is described in (Lukovic et al., 2019). Alignment of the sequencing reads to the *Sus scrofa* reference genome assembly (Swine Genome Sequencing Consortium Sscrofa10.2/susScr3 UCSC) (Archibald et al., 2010), feature counting for the corresponding reference annotation, and the statistical analysis of differential expression were conducted by the TopHat-Cufflinks workflow as described in (Trapnell et al., 2012). For this analysis, TopHat version 2.1.1 (Kim et al., 2013), Bowtie2 version 2.2.3 (Langmead and Salzberg, 2012), and Cufflinks version 2.2.1 (Trapnell et al., 2010) were used.

#### Human Heart Samples

Raw reads assessed by RNA-sequencing of the human samples were preprocessed by Cutadapt (version 1.15) (Martin, 2011). In this step adapters, the poly(A) tail and bases below Phred score 30 were trimmed, and reads with a length of less than 19 nt were filtered out (Williams et al., 2016). Quality control analysis was performed using the FastQC (version 0.11.8) and MultiQC (version 1.7) (Ewels et al., 2016) software. HISAT2 (version 2.0.4) (Kim et al., 2019), featureCounts (version of Subread 2.0.0) (Liao et al., 2014), and DESeq2 (version 1.10.1) (Love et al., 2014) were utilized for alignment, annotation, normalization, and differential expression analysis, respectively. *Homo sapiens* Ensembl GRCh37 reference genome and annotation were used for the analysis of the human samples (Yates et al., 2016). Differential expression analysis was conducted by the DESeq2 software package (Love et al., 2014).

### RNA Scope^®^ *In Situ* Hybridization

RNA Scope^®^ *in situ* hybridization assay was performed on left ventricle tissue slides harvested from human control hearts (the same study as described previously: IK-NPIA-0021–14/1426/18 project 4) using RNA Scope^®^ Multiplex Fluorescent Kit v2 according to the manufacturer’s instructions (Advanced Cell Diagnostics Pharma Assay Services, Newark, CA, United States). Briefly, 4 µm formalin-fixed paraffin-embedded tissue sections were pretreated with heat and protease prior to hybridization with the following target oligo probes: 3plex-Hs-Positive Control Probe (catalog number: 320861), 3plex-Hs-Negative Control Probe (catalog number: 320871), Hs-SSTR1-C1 (catalog number: 310581, accession no.: NM_001049.2), Hs-SSTR2-C1 (catalog number: 310571, accession no.: NM_001050.2), Hs-TAGNL-C3 (catalog number: 498961-C3, accession no.: NM_003186.3), Hs-PECAM1-O1-C3 (catalog number: 487381-C3, accession no.:. NM_000442.4), and Hs-RYR2-C2 (catalog number: 415831-C2, accession no.: NM_001035.2; see also Supplementary Table S2 for the list of probes). Cell type-specific markers were used to identify cardiomyocytes with a probe recognizing the mRNA of Ryanodine receptor 2 (*RYR2*) (Lanner et al., 2010), endothelial cells with a probe recognizing the mRNA of platelet endothelial cell adhesion molecule 1 (*PECAM-1*) (Feng et al., 2004), and vascular smooth muscle cells with a probe recognizing the mRNA of Transgelin (*TAGLN*) (Camoretti-Mercado et al., 1998), respectively. Preamplifier, amplifier, and AMP-labeled oligo probes were then hybridized sequentially, followed by chromogenic precipitate development. Each sample was quality controlled for RNA integrity with a positive control probe specific to the housekeeping genes with a negative control probe. The pretreatment conditions were optimized to establish the maximum signal-to-noise ratio. Specific RNA staining signal was identified as red/green punctate dots. Nuclei were stained with 4’,6-diamidino-2-phenylindole (DAPI) appearing light purple. Imaging was performed with Leica DMI8 Confocal microscope.

### Gene Ontology (GO) Enrichment Analysis

GO enrichment analysis (database version released on August 10, 2020) was performed for each possible comparison of the experimental groups. To obtain GO biological process terms enriched among differentially expressed genes compared to the *Sus scrofa* reference gene list, online PANTHER Overrepresentation Test [geneontology.org, version released on July 28, 2020 (Mi et al., 2019)] was utilized. Enrichment analysis was conducted by applying Fisher’s exact test with false discovery rate adjustment for multiple comparisons (Benjamini and Hochberg, 1995).

### Statistical Analysis

Statistical analyses and Rout outlier analyses were performed and graphs were created using GraphPad Prism version 8 (GraphPad Software, San Diego, CA, United States). The Kruskal-Wallis test with Dunn’s post hoc test, one-way analysis of variance (ANOVA), and unpaired *t*-test were used to find statistically significant differences. Differences were considered significant at values of *p* < 0.05 (*p* values are indicated as *p < 0.05; **p < 0.01; ***p < 0.001). Unless noted otherwise, all data represent the mean ± SEM. To avoid the possibility of overlooking significant differences due to small group sizes, ANOVA-like nonparametric bootstrap-based comparison of means with 1,000 times resampling (Davison and Hinkley, 1997) was also performed on relative expression ratios assessed by RT-qPCR.

## Results

### Protective Effect of SST on H9C2 and Adult Rat Cardiomyocyte Cell Viability Subjected to Simulated Ischemia/Reperfusion

In order to investigate the cardiocytoprotective effect of SST, *in vitro* simulated ischemia/reperfusion experiments were performed in H9C2 cells and adult rat cardiomyocytes, respectively. Simulated ischemia/reperfusion significantly decreased the H9C2 and adult rat cardiomyocyte cell viability (Supplementary Figure S1) compared to normoxic controls. The simulated ischemia/reperfusion-induced cell death was reversed by SST treatment at 100 and 300 nM (Figure 1C) showing a concentration-dependent, bell-shaped cardiocytoprotective effect in H9C2 cells. SST did not influence the viability of normoxic cells (Figure 1B), except for the highest concentration of SST (1 µM) that caused a significant increase of cell death in H9C2 cells. SST also shows a concentration-dependent, bell-shaped cardiocytoprotective effect against simulated ischemia/reperfusion-induced injury in adult rat cardiomyocytes (Figure 2C) where SST treatment at 1, 10, 100, and 300 nM concentrations increased the viability significantly. Although the cell viability shows a similar dose-response pattern in the SST-treated groups in normoxic conditions (Figure 2B), these changes were not statistically significant.

### SST-Like Immunoreactivity in the Porcine Left Ventricle and Plasma

To determine SST-like immunoreactivity in pig plasma and left ventricle samples, RIA was performed. SST-like immunoreactivity was detected in pig left ventricle samples, but no differences were determined between groups (Figure 3C). Significantly increased plasma SST-like immunoreactivity was measured in ischemic preconditioning samples compared to the sham group (Figure 3B).

### *SSTR1* and *SSTR2* mRNA Expression in Porcine Left Ventricle

**FIGURE 4:**
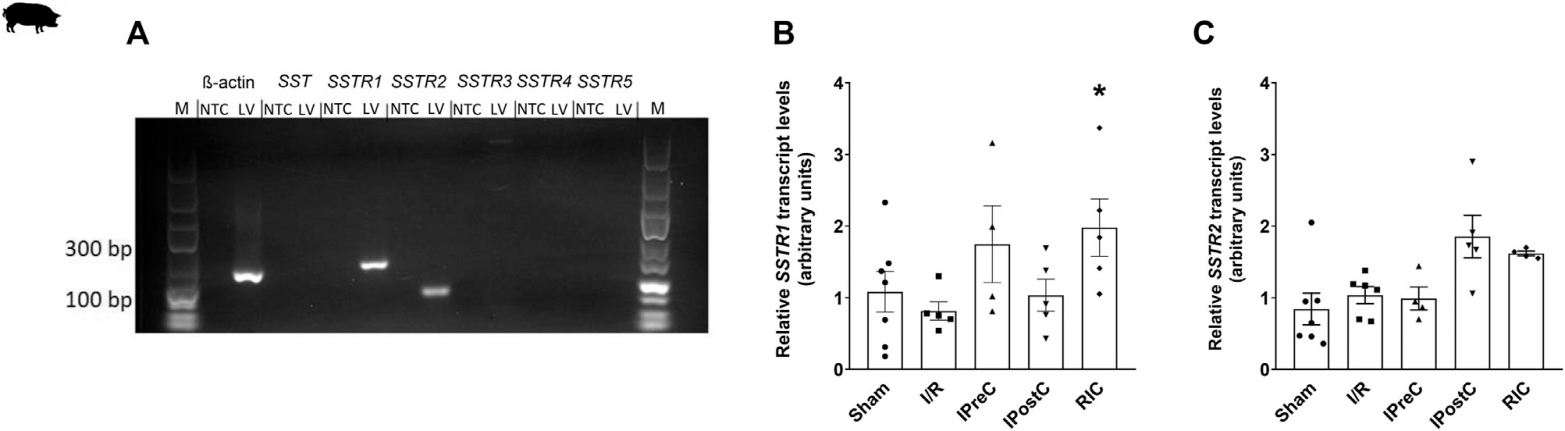
Expression of somatostatin and its receptors in porcine left ventricle samples **(A)**. The outer lanes contain a DNA ladder (M). No template control (NTC) samples without cDNA did not give any amplification products with the used primers, indicating that contamination was not present. Product sizes: β-actin: 133 bp; SST: 117 bp; SSTR1: 139 bp; SSTR2: 82 bp; SSTR3: 110 bp; SSTR4: 112 bp; SSTR5: 98 bp. Relative gene expression ratios of SSTR1 **(B)** and SSTR2 **(C)** in left ventricle samples. Transcript levels were normalized to ß-actin. Data are presented as mean ± SEM. The Kruskal-Wallis test and Dunn’s post hoc test, *p < 0.05 vs. I/R SSTR1–5: somatostatin receptors one to five, I/R: ischemia-reperfusion (SSTR1: n = 5, SSTR2: n = 6), IPreC: ischemic preconditioning (n = 4), IPostC: ischemic postconditioning (n = 5), and RIC: remote ischemic conditioning (SSTR1: n = 5, SSTR2: n = 4) and sham-operated group (n = 7).

In order to detect *SST* and its receptor mRNA expression in pig left ventricle, RT-qPCR and bioinformatics analysis of mRNA-sequencing data were performed. We also performed a bootstrap-based comparison of means to confirm the RT-qPCR results. Expression of *SSTR1* and *SSTR2* mRNA was detected by PCR, but not *SST* and its other receptors (*SSTR3*, *SSTR4*, and *SSTR5*) (Figure 4A). The results of the bioinformatics analysis of mRNA-sequencing data confirmed all these results (Table 1). The relative expression of *SSTR1* was significantly upregulated in the remote ischemic conditioning group compared to the ischemia/reperfusion group (Figure 4B). There were no significant differences in *SSTR2* mRNA expression between any groups (Figure 4C). These results were also confirmed by bootstrap analysis.

**TABLE 1:** Expression of *SST* and its receptors in porcine left ventricular samples (sham group) measured by mRNA sequencing.

Gene	FPKM
*SST*	Undetectable
*SSTR1*	0.3881
*SSTR2*	0.3571
*SSTR3*	Undetectable
*SSTR5*	Undetectable

LV: left ventricle, SST: somatostatin, *SSTR1–5*: somatostatin receptors one to five, and FPKM: fragments per kilobase of transcript per million mapped reads.

### SST-Like Immunoreactivity in the Human Interventricular Septum

**FIGURE 5:**
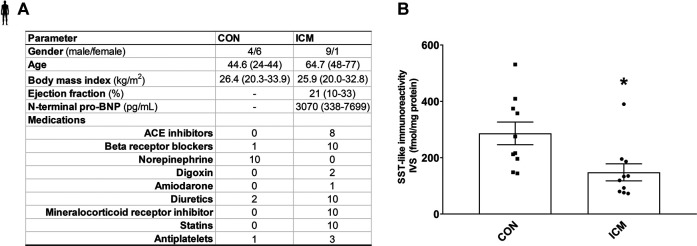
Control and ICM patient’s characteristics **(A)**. SST-like immunoreactivity of interventricular septum samples **(B)**. Data are presented as mean ± SEM. unpaired *t*-test *p < 0.05 vs. CON (n = 10–10), ICM: ischemic cardiomyopathy, and CON: control.

To determine SST-like immunoreactivity in human interventricular septum samples, RIA was performed. The patient’s clinical characteristics are described in Figure 5A. A significant decrease of tissue SST-like immunoreactivity was measured in ICM samples compared to the control group (Figure 5B).

### *SSTR1, SSTR2,* and *SSTR5* mRNA Expression in Human Left Ventricle

To detect *SST* and its receptor mRNA expression in human left ventricles bioinformatics analysis of the mRNA-sequencing data was performed. The expression of the mRNA of *SSTR1, SSTR2,* and *SSTR5* receptors was detected, but not *SST* and its other receptors (*SSTR3* and *SSTR4*) (Table 2).

**TABLE 2:** Expression of SST and its receptors in human left ventricular samples measured by mRNA sequencing.

Gene	Normalized read count
*SST*	Undetectable
*SSTR1*	3.950
*SSTR2*	8.402
*SSTR3*	Undetectable
*SSTR4*	Undetectable
*SSTR5*	49.064

LV: left ventricle, SST: somatostatin, SSTR1–5: somatostatin receptors one to five.

### Localization of *SSTR1* and *SSTR2* mRNA in Vascular Endothelial Cells of the Human Left Ventricle

**FIGURE 6:**
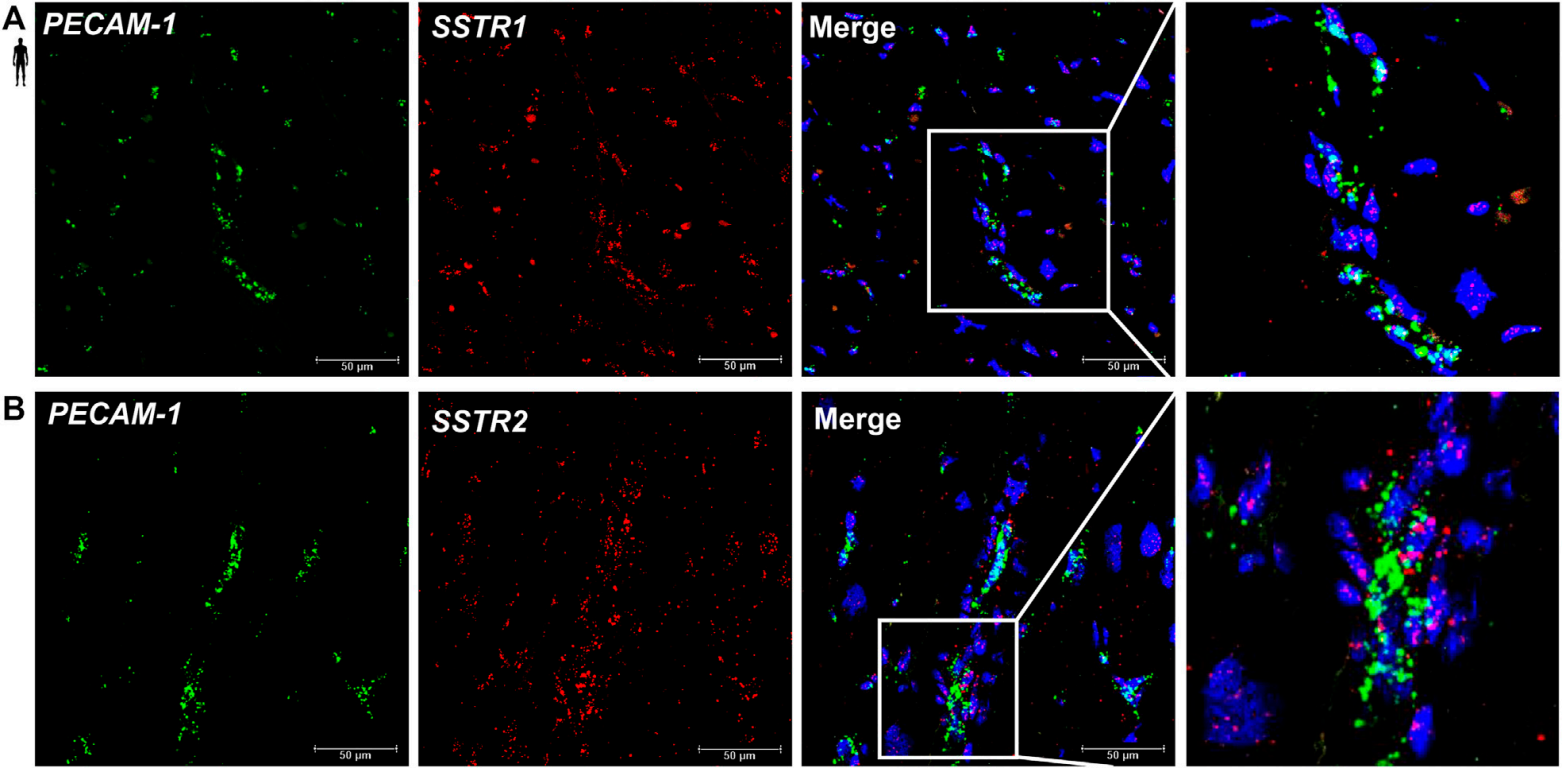
Representative confocal microscopy images of RNA Scope®-*SSTR1* **(A)** and *SSTR2* **(B)** mRNA expression in histological samples of human control left ventricle. Nuclei were stained with DAPI (blue). Fluorescein-labeled tyramide (green) was used to visualize mRNA of *PECAM-1* (endothelial marker) and cyanine 3- (Cy3-) labeled tyramide (red) was used to visualize mRNA of *SSTR1* or *SSTR2*, respectively. Scale bar represents 50 µm.

**FIGURE 7:**
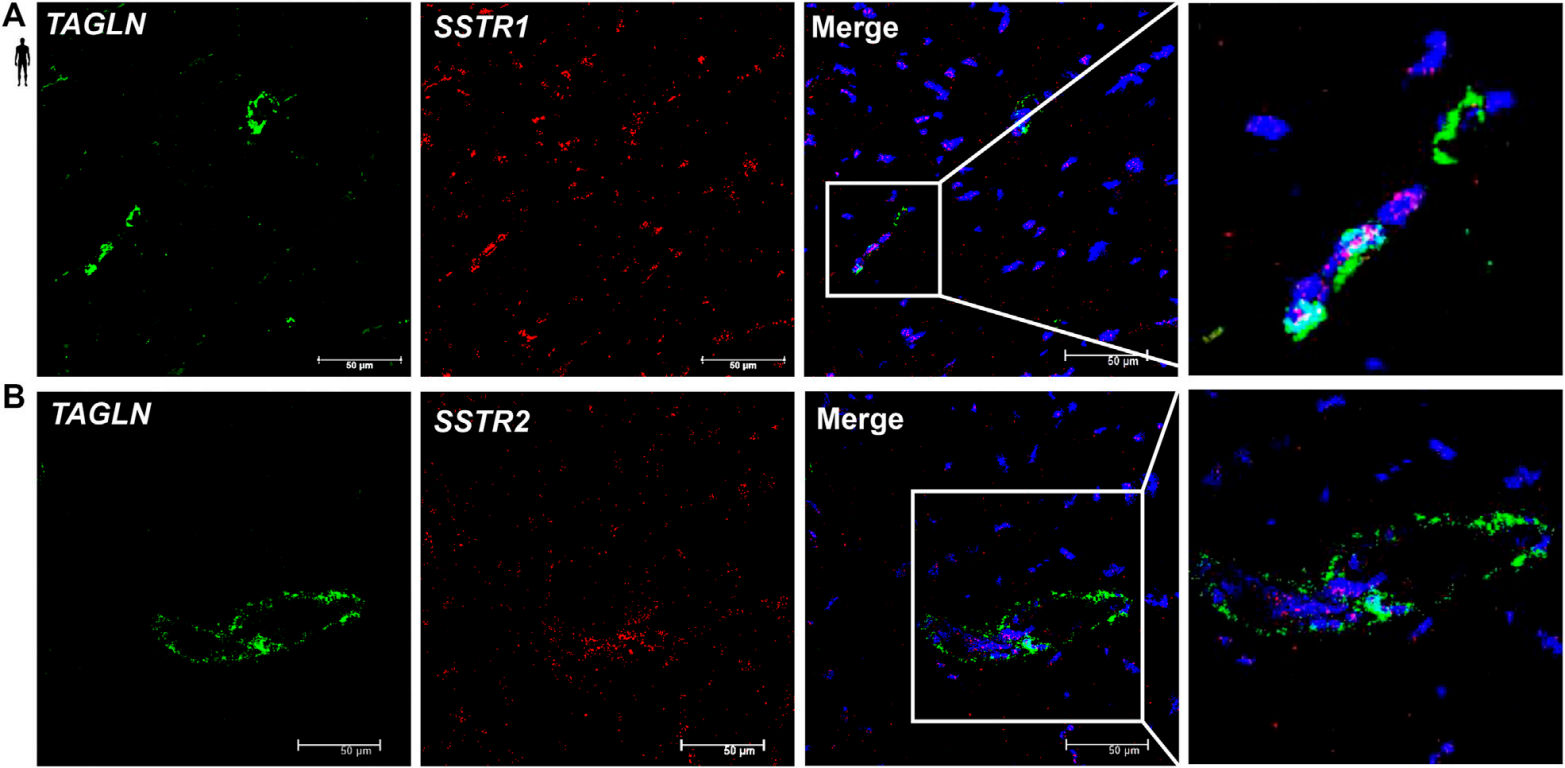
Representative confocal microscopy images of RNA Scope®-*SSTR1* **(A)** and *SSTR2* **(B)** mRNA expression in histological samples of human control left ventricle. Nuclei were stained with DAPI (blue). Fluorescein-labeled tyramide (green) was used to visualize mRNA of *TAGLN* (smooth muscle marker) and Cy3-labeled tyramide (red) was used to visualize mRNA of *SSTR1* or *SSTR2*, respectively. Scale bar represents 50 µm.

**FIGURE 8:**
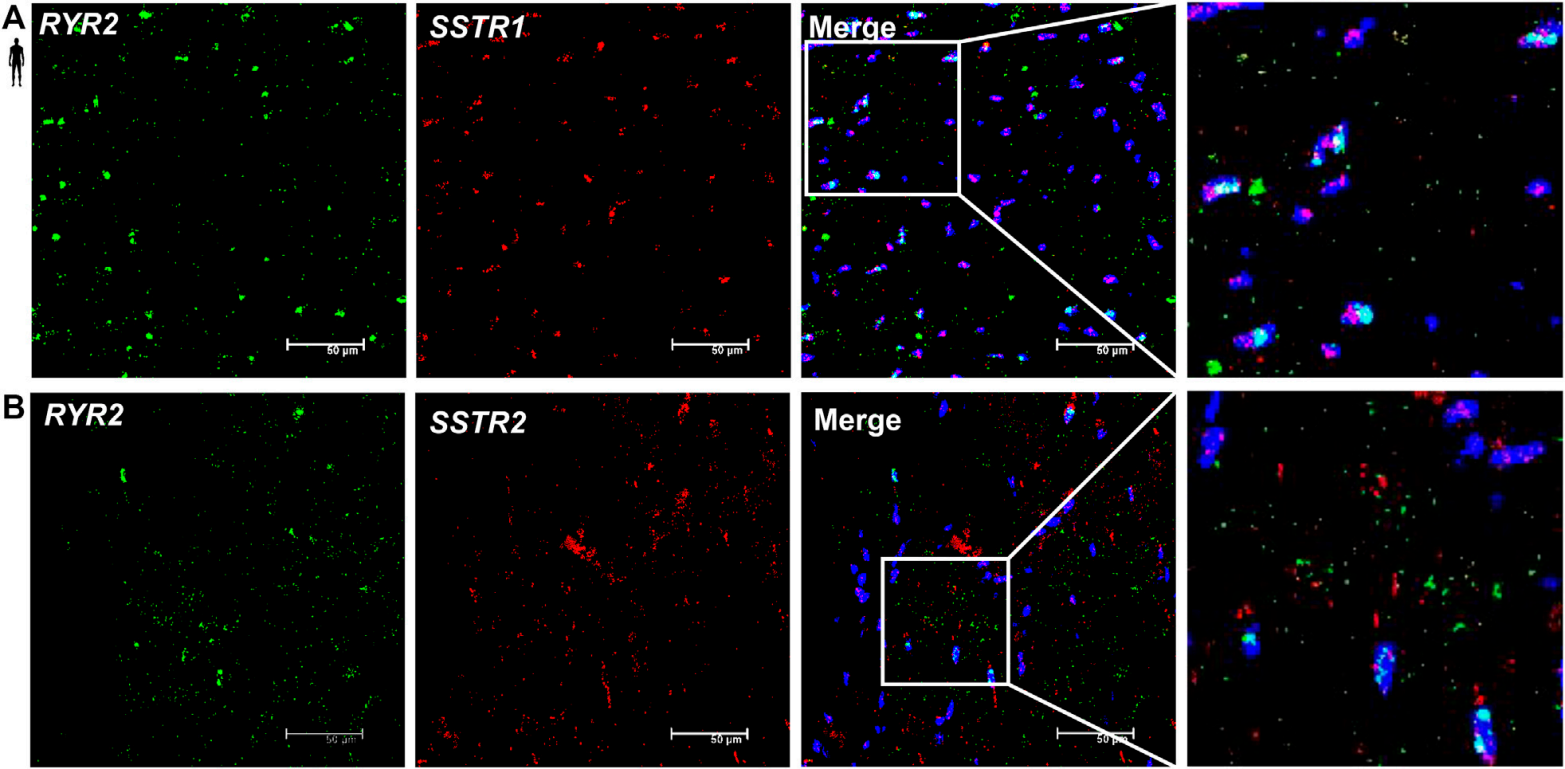
Representative confocal microscopy images of RNA Scope®-*SSTR1* **(A)** and *SSTR2* **(B)** mRNA expression in histological samples of human control left ventricle. Nuclei were stained with DAPI (blue). Fluorescein-labeled tyramide (green) was used to visualize mRNA of *RYR2* (myocardial marker) and Cy3-labeled tyramide (red) was used to visualize mRNA of *SSTR1* or *SSTR2*, respectively. Scale bar represents 50 µm.

To determine the cell type-specific localization of mRNA of *SSTR1 and SSTR2* in human healthy left ventricular samples, RNA Scope^®^ *In Situ* Hybridization assay was performed (Figures 6–8). Expression of mRNA of both *SSTR1* and *SSTR2* was shown primarily in *PECAM-1* mRNA-positive endothelial cells (Figures 6A,B, respectively); however, both *SSTR1* and *SSTR2* mRNA were detected in other cell types, including *TAGLN* mRNA-positive vascular smooth muscle cells (Figure 7) and *RYR2* mRNA-positive cardiomyocytes (Figure 8). There was no detectable signal on the negative control slides (Supplementary Figure S2).

### GO Analysis of mRNA-Sequencing Data

**FIGURE 9:**
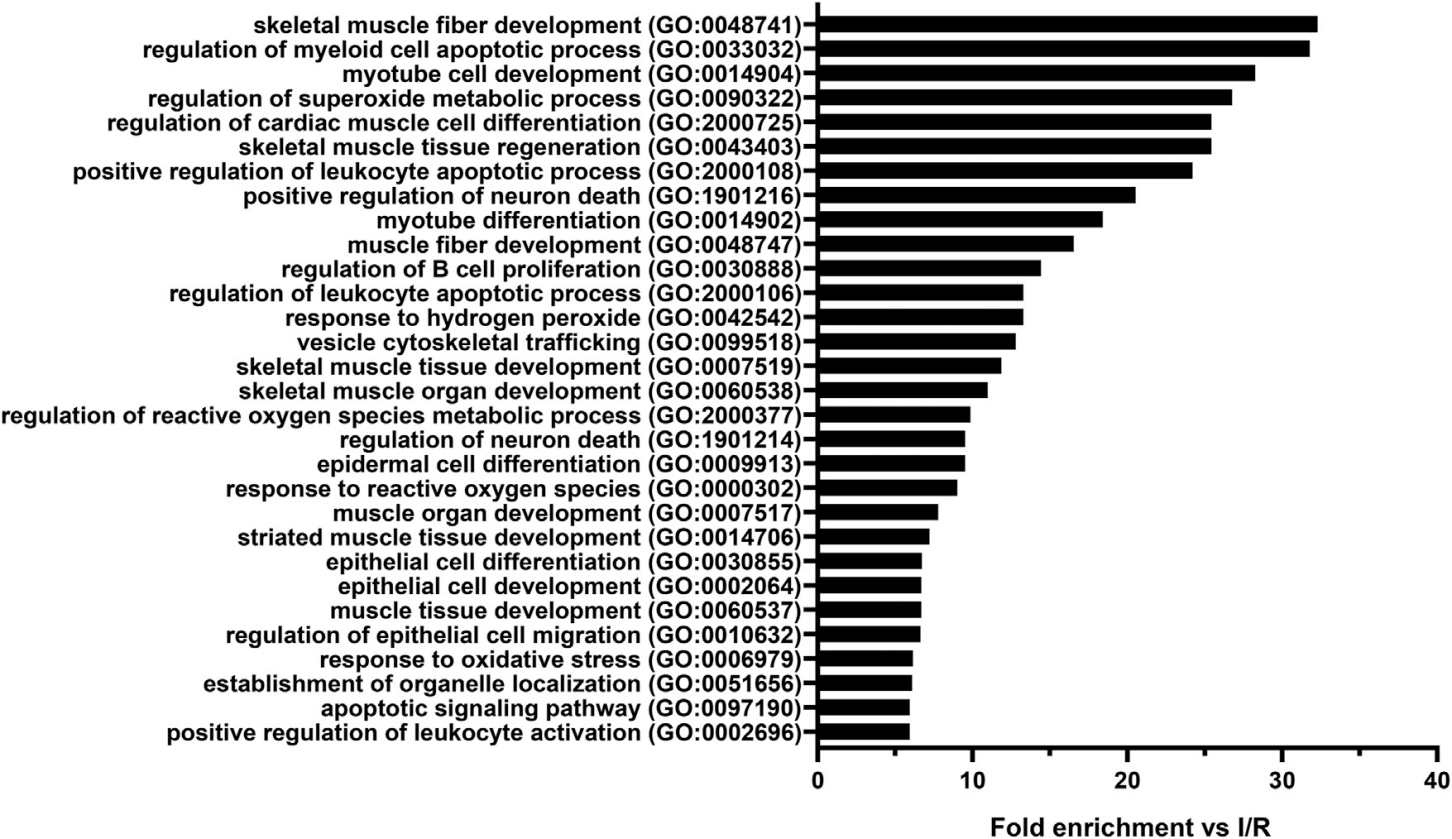
Top thirty Gene Ontology (GO) biological process terms significantly enriched among the mRNAs (n = 143) differentially expressed in the comparison between remote ischemic conditioning (RIC) and ischemia/reperfusion (I/R) groups in the order of decreasing fold enrichment values. GO enrichment analysis highlights the effect of RIC on skeletal muscle fiber development, muscle tissue regeneration, cardiac muscle cell differentiation, superoxide metabolic processes, reactive oxygen species metabolic processes, and response to oxidative stress. Adjusted Fisher’s exact *p* values were <0.05 in case of each shown process after false discovery rate adjustment for multiple comparisons.

To identify the biological processes that could play a role in the cytoprotective effect of SST, GO analysis of all differentially expressed mRNAs was performed for each possible comparison of the experimental groups. The results of the comparison between remote ischemic conditioning and ischemia/reperfusion groups—where *SSTR1* expression significantly increased—are presented (Supplementary Table S3). Differentially expressed mRNAs were significantly associated with, e.g., cardiac muscle differentiation, skeletal muscle development/regeneration, and response to oxidative stress (Figure 9).

## Discussion

Here we showed that SST protects rat cardiomyocytes against ischemia/reperfusion injury. Moreover, in a translational acute myocardial infarction pig model, ischemic preconditioning increased plasma SST-like immunoreactivity, and in the left ventricle, it was detectable at the peptide, but not at the mRNA level. *SSTR1* and *SSTR2* mRNAs were expressed in the pig left ventricle, and remote ischemic conditioning upregulated *SSTR1*. In healthy humans, left ventricular samples *SSTR1*, *SSTR2*, and *SSTR5* mRNAs were expressed, and *SSTR1* and *SSTR2* mRNAs were located in vascular endothelial cells. Moreover, SST expression was decreased at the peptide level in interventricular septum samples of patients with ischemic cardiomyopathy. These results show for the first time that SST exerts a direct cardioprotective effect against simulated ischemia/reperfusion injury. Moreover, SST is expressed in the heart tissue at the peptide level; however, it is likely to be of sensory neural origin since its mRNA is not detectable. SSTR1 and SSTR2 might be involved in the cardioprotective action of SST; however, other non-SSTR-mediated mechanisms cannot be excluded.

We provided here the first evidence that native STT exerts a concentration-dependent direct cardiocytoprotective effect against simulated ischemia/reperfusion injury in adult rat cardiomyocytes and H9C2 cells. This finding is supported by earlier data showing that SST protects against retinal ischemia/reperfusion injury in a mouse model (Wang et al., 2017) and implies that SST may have a general anti-ischemic effect in a variety of cells. All *Sstrs* were described in both rat cardiomyocytes and H9C2 cell lines, although only the expression of *Sstr3, Sstr4,* and *Sstr5* was abundant (Granata et al., 2004; Bell et al., 2008). Accordingly, SST and a related peptide, cortistatin-14, and the synthetic SSTR2, SSTR3, and SSTR5 agonist octreotide significantly reduce brain infarct size in a middle cerebral artery occlusion model (Rauca et al., 1999). Moreover, octreotide protects against high intraocular pressure-induced retina ischemia/reperfusion damage in a mouse model (Wang et al., 2015), reduces ischemia/reperfusion injury in rat pancreas and in rabbit liver (Hoffmann et al., 1996; Yang et al., 2013) and reduces infarct size in a coronary occlusion model in rats (Wang et al., 2005). It is worth mentioning that, according to the results of a recent clinical trial, long-acting octreotide treatment shows low risk for cardiac adverse events in patients with diabetic retinopathy (Pivonello et al., 2018). Another synthetic analog of SST, pasireotide, also protects against chemically induced ischemia/reperfusion injury *ex vivo* in a rat retina (Kokona et al., 2012).

After demonstrating the direct cardiocytoprotective effect of the native SST *in vitro,* we aimed to obtain further data supporting the protective role of SST in a translational pig model of myocardial infarction and cardioprotection, as well as demonstrate the expression of the different SST receptors. Therefore, here, we used tissue and plasma samples obtained from our previous pig myocardial infarction and cardioprotection study with different ischemic conditioning interventions (Baranyai et al., 2017). In this model, plasma SST-like immunoreactivity increased after ischemic preconditioning, when a significant infarct size reduction was seen (Baranyai et al., 2017), suggesting the potential role of SST in this protective action. In heart failure patients (left ventricular ejection fraction 18 ± 8%, NYHA classes III-IV), the plasma SST concentration was found to be around 18 pmol/l (Deis et al., 2020), which is a similar range found in our present study in porcine plasma showing that human and the porcine plasma SST level might be in the same range. Another important finding of our present study is that SST-like immunoreactivity was detected in the pig left ventricle, but without the expression of its mRNA. Therefore, one may speculate that SST in the heart tissue is likely to be of sensory neural origin. TRPV1 receptors expressed on the peptidergic sensory fibers innervating the heart (Randhawa and Jaggi, 2015) can be activated by mediators of tissue injury and inflammation, such as protons and prostaglandins leading to sensory neuropeptide release (Bencsik et al., 2020) including SST. Although our present study provided the first data for the presence of SST in the heart, earlier data showed elevated plasma CGRP concentration in a rat model of ischemia/reperfusion after ischemic preconditioning, which can also mediate cardioprotection (Chen et al., 1999; Luo et al., 2004; Randhawa and Jaggi, 2015). In addition, in the present study, we demonstrated the expression of *SSTR1* and *SSTR2* in the pig left ventricular heart tissue samples by both RT-qPCR and deep sequencing. Here, we also showed that remote ischemic conditioning upregulated *SSTR1* measured by RT-qPCR. It is important to note that different conditioning approaches exert a different type of cardioprotective effects, e.g., infarct size reduction in ischemic preconditioning and vasculoprotective effects (reduction in edema) in ischemic postconditioning and remote ischemic conditioning (Baranyai et al., 2017). This may explain the differences in SST or SSTR expressions in the different experimental groups. These results show that cardioprotection of SST might be mediated by SSTR1 and SSTR2; however, other mechanisms cannot be excluded.

We also aimed to reveal the potential biological processes contributing to the protective mechanism of SST, SSTR1, and SSTR2 using the GO analysis of the mRNA-sequencing data. The GO analysis revealed that potential receptor-linked mechanisms include muscle development, differentiation, and regeneration, as well as apoptosis regulation, but nonreceptor-mediated protective mechanisms of SST are also possible, such as oxidative stress, reactive oxygen species regulation, and metabolic processes as suggested by literature data (Yang et al., 2009; Solarski et al., 2019). Accordingly, experimental data show that the inhibition of Na^+^/H exchanger, Rho/Rac, and e-NOS pathways as well as the activation of protein tyrosine phosphatase and MAPK/ERK pathway can be related to SSTR1 signaling, while Ca^2+^ channel, inward-rectifying K^+^ current, e-NOS inhibition, and protein tyrosine phosphatase activation might be linked to SSTR2 signaling (Rai et al., 2015).

Finally, to further study the translational relevance of the experimental findings in cells and pig hearts, we performed the highly sensitive RNA Scope^®^ *in situ* hybridization method for SST receptors in histological section of healthy human heart tissue samples and showed the expression of *SSTR1* and *SSTR2* in vascular endothelial cells and cardiomyocytes. However, due to the diffuse pattern of RNA *in situ* hybridization signal, we cannot rule out *SSTR1* and *SSTR2* expression by other cell types of the myocardium. In addition, here, we demonstrated the expression of *SSTR1*, *SSTR2*, and *SSTR5* in human left ventricular heart tissue samples by deep sequencing. We also measured SST-like immunoreactivity in interventricular septum samples of healthy and ischemic cardiomyopathy patients and demonstrated the first time in the literature that SST is expressed in the human heart tissue at the peptide level. Cardiac SST showed a decreased expression at the peptide level in the tissue samples of patients with ischemic cardiomyopathy as compared to controls. In line with our present findings, octreotide treatment significantly improved cardiac function in eight patients with ischemic cardiomyopathy (NYHA class III, ejection fraction < 40%) (Eryol et al., 2004). Our present finding that *SSTR1*, *SSTR2*, and *SSTR5* are expressed at the mRNA level in human left ventricle are supported by earlier data showing the mRNA expression of *SSTR1, SSTR2, SSTR4,* and *SSTR5,* but not *SSTR3* in the human atria and ventricle (Smith et al., 2005). In the study of Smith et al., 2005, cultured human fibroblasts expressed *SSTR1, SSTR2, SSTR4,* and *SSTR5* receptors, but cardiomyocytes only *SSTR1* and *SSTR2*; however, vascular endothelial cells were not investigated (Smith et al., 2005). These results suggest that SSTR1 and SSTR2 might also be an important contributor to the cardioprotective effect of SST; however, other mechanisms cannot be excluded.

## Conclusion

Our present results provide the first evidence that SST protects cardiomyocytes against ischemia/reperfusion injury. Moreover, SST is expressed in the heart tissue at the peptide level; however, it is likely to be of sensory neural origin since its mRNA is not detectable. SSTR1 and SSTR2 might be involved in the cardioprotective action of SST, but other mechanisms cannot be excluded. These results open new perspectives on the pharmacological relevance of SST and SSTR signaling in cardioprotection.

### Limitations

*SSTR4-*specific primer pairs were designed using a partial coding sequence. The available mRNA sequence of pig *SSTR4* is not covering the complete region encoding the protein sequence from start to stop codon. Furthermore, the corresponding *SSTR4* gene is missing from the used *Sus scrofa* reference annotation; therefore, it could not be measured by RNA-sequencing. Furthermore, we could not provide functional evidence for the SSTR receptor-mediated mechanisms due to the lack of selective receptor antagonists.

## Data Availability

The datasets presented in this study can be found in online repositories. The names of the repository/repositories and accession number(s) can be found at E-MTAB-10720.

## Glossary

ANOVA: analysis of variance
BSA: bovine serum albumin
cDNA: complementary DNA
CGRP: calcitonin gene-related peptide
Cy3: cyanine 3
DAPI: 4′,6-diamidino-2-phenylindole
D-PBS: Dulbecco’s phosphate-buffered saline
e-NOS: endothelial nitric oxide synthase
ERK: extracellular signal-regulated kinase
FBS: fetal bovine serum
FPKM: fragments per kilobase of transcript per million mapped reads
GO: Gene Ontology
ICM: ischemic cardiomyopathy
LAD: left anterior descending coronary artery
LV: left ventricle/left ventricular
MAPK: mitogen-activated protein kinase
mRNA: messenger RNA
NTC: no template control
PACAP: pituitary adenylate cyclase-activating polypeptide
PECAM-1: platelet endothelial cell adhesion molecule 1
Rac: Ras-related C3 botulinum toxin substrate
RFU: relative fluorescent unit
Rho: Ras homolog family member
RIA: radioimmunoassay
RT-qPCR: real-time quantitative polymerase chain reaction
RYR2: Ryanodine receptor 2
SEM: standard error of the mean
SP: substance P
SST: somatostatin
SSTR1-5: somatostatin receptors 1–5
TAGLN: transgelin
TRPV1: Transient Receptor Potential Vanilloid 1
