# Supplementary material for: Somatostatin and Its Receptors in Myocardial Ischemia/Reperfusion Injury and Cardioprotection
Source: Front Pharmacol. 2021 Nov 5;12:663655. doi: 10.3389/fphar.2021.663655 (PMC8602362; doi:10.3389/fphar.2021.663655)
Supplement: Supplementary file 1 [file Presentation1.pdf]

Supplementary material

H9C2

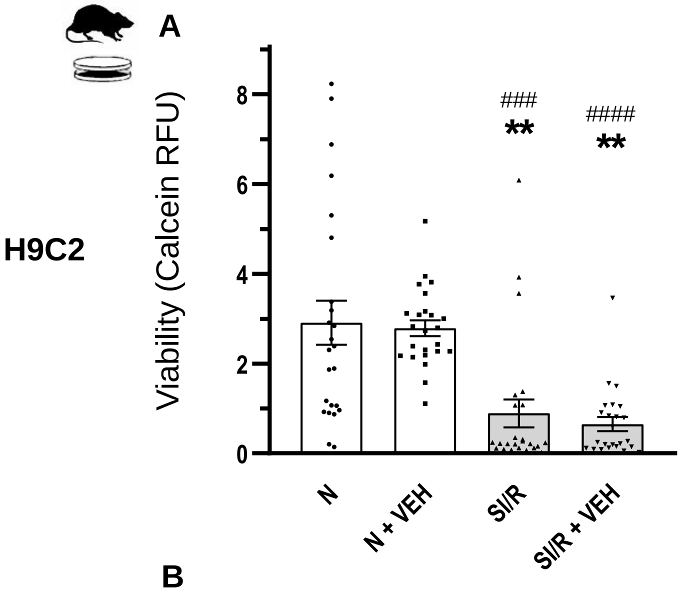

Comparisons	Summary	p-value
**N vs. N + VEH**	ns	>0.9999
**N vs. SI/R**	**	0.0045
**N vs. SI/R + VEH**	**	0.0013
**N + VEH vs. SI/R**	###	0.0003
**N + VEH vs. SI/R + VEH**	####	<0.0001
**SI/R vs. SI/R + VEH**	ns	>0.9999

ARCM

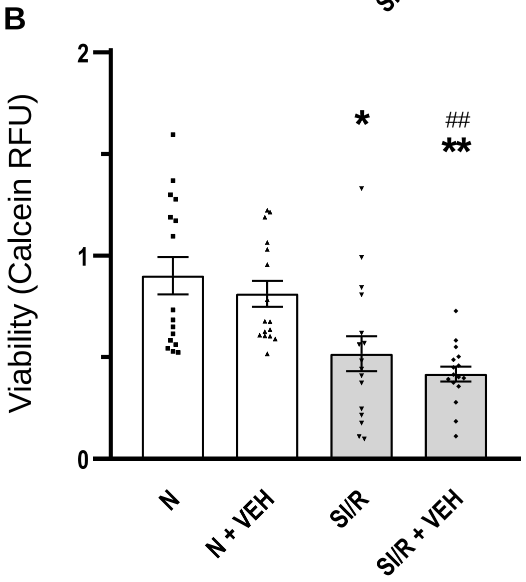

Comparisons	Summary	p-value
**N vs. N + VEH**	ns	>0.9999
**N vs. SI/R**	*	0.0376
**N vs. SI/R + VEH**	**	0.0017
**N + VEH vs. SI/R**	ns	0.0628
**N + VEH vs. SI/R + VEH**	##	0.0032
**SI/R vs. SI/R + VEH**	ns	>0.9999

Figure S1. Viability of H9C2 (**A**) cells and adult rat cardiomyocytes (ARCM) (**B**) after in vitro simulated ischemia-reperfusion experiment. Data are presented as mean ± SEM. Kruskal-Wallis test, Dunn's post hoc test $^{*/\#}p<0.05$ $^{**/\#\#}p<0.01$, $^{***/\#\#\#}p<0.001$, $^{****/\#\#\#\#}p<0.0001$. N: Normoxia, VEH: vehicle, SI/R: simulated ischemia/reperfusion, RFU: relative fluorescence unit.

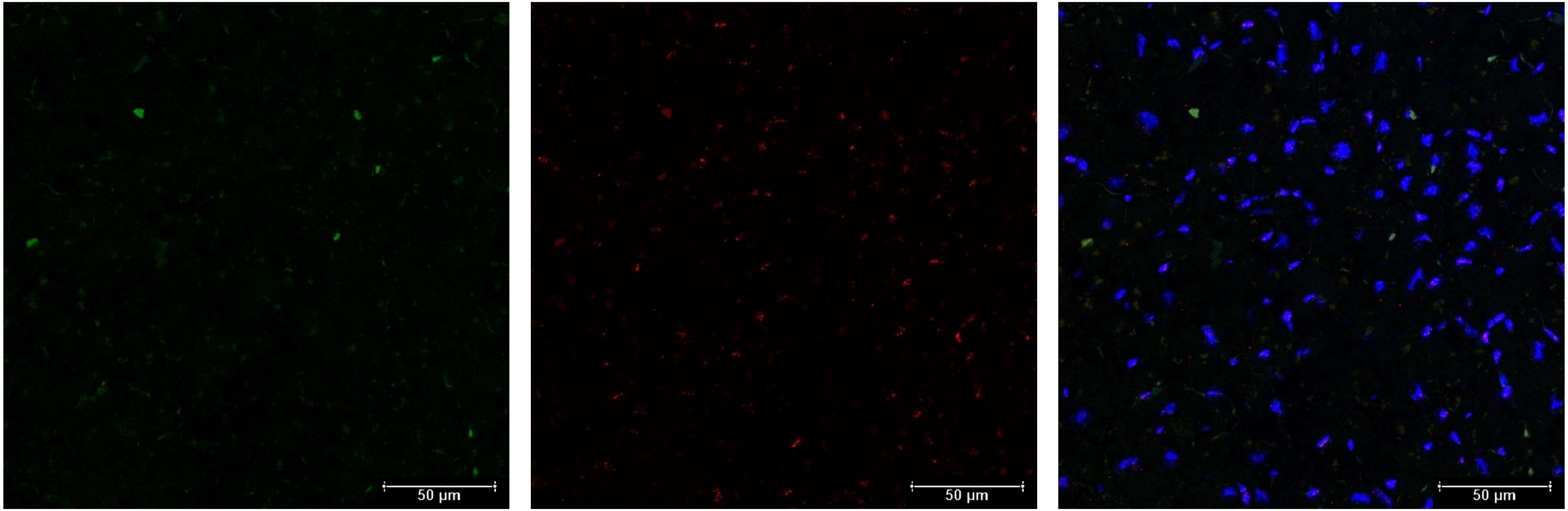

Figure S2. Representative Confocal Microscopy Images of RNA Scope® negative control (green, red) in histological samples of human control left ventricle. Cells were counterstained with DAPI (blue).

Gene	Accession number	Forward primer	Reverse primer	Product size (bp)
SST	NM_001009583.1	CTCTCCATCGTCCTGGCTCT	GTACTTGGCCAGTTCCTGCT	117
SSTR1	NM_001190231.1	CATTCTGCCCATCGTGGTCT	CAGCAGGAAGCCCATGAGAA	139
SSTR2	NM_001011694.1	GAATCCGAGTGGGTTCCTCC	ATGAAGACGGCCACCACAAT	82
SSTR3	NM_001167628.1	TCATCAATGTGGTGTGCCCT	GCCATAGAGGATGGGGTTGG	110
SSTR4	AY156053.1	GCTGAACCTCTTCGTGACCA	GTTGTCGGAGAGGAAGCCAT	112
SSTR5	NM_001038229.1	CAGAACGCCCTCTCCTACTG	GTGAGGCAGAAGATGCTGGT	98
β-actin	XM_003124280.5<br>XM_021086047.1	CGGCATCCACGAAACTACCT	CTCCTTCTGCATCCTGTCGG	133

Table S1. Details of oligonucleotide primer sequences used in qRT-PCR. *SST*: somatostatin; *SSTR1-5*: somatostatin receptor type 1-5; bp: base pair.

Probe name	Catalogue number	Dye
3plex-Hs-Positive Control Probe	320861	Cyanide 3, FITC
3plex-Hs-Negative Control Probe	320871	Cyanide 3, FITC
Hs-SSTR1-C1	310581	Cyanide 3
Hs-SSTR2-C1	310571	Cyanide 3
Hs-TAGNL-C3	498961-C3	FITC
Hs-PECAM1-O1-C3	487381-C3	FITC
Hs-RYR2-C2	415831-C2	FITC

Table S2. Probes and dyes used in RNA Scope® experiment for determination of the localization of mRNAs of interest. SSTR1, somatostatin receptor type 1; SSTR2, somatostatin receptor type 2; TAGLN, transgelin; PECAM1, platelet and endothelial cell adhesion molecule 1; RYR2, Ryanodine receptor 2.
